# CAFs-derived small extracellular vesicles circN4BP2L2 promotes proliferation and metastasis of colorectal cancer via miR-664b-3p/HMGB3 pathway

**DOI:** 10.1080/15384047.2022.2072164

**Published:** 2022-06-19

**Authors:** Keda Yang, Fan Zhang, Baihua Luo, Zhan Qu

**Affiliations:** a Department of Pathology, Xiangya Hospital, Central South University, Changsha, Hunan Province, China; b Department of Gynecology, Xiangya Hospital, Central South University, Changsha, Hunan Province, China; c Department of General Surgery, Xiangya Hospital, Central South University, Changsha, Hunan Province, China

**Keywords:** Colorectal cancer, cancer-associated fibroblasts, small extracellular vesicle (sEV), proliferation, migration, circN4BP2L2

## Abstract

Our previous research has demonstrated that colorectal cancer (CRC) progression was promoted by circN4BP2L2. This study aimed to further explore the mechanism of circN4BP2L2 in the development of CRC from the perspective of small extracellular vesicles (sEVs). Cancer-associated fibroblasts cell (CAFs) and normal fibroblasts cell (NFs) were isolated from CRC tissues and adjacent tissues, respectively. The ultra-centrifugation was used for extraction of their related sEVs. Cell proliferation and apoptosis were analyzed using CCK-8 and flow cytometry, respectively. Transwell assay was conducted to measure cell migration. The tube formation ability was assessed by tube formation assay. The target relationships between circN4BP2L2 and miR-664b-3p, and miR-664b-3p and HMGB3 were validated by dual-luciferase reporter detection. The effect of CAFs-derived sEV (CAFs-sEVs) circN4BP2L2 on CRC was further studied in nude mice. CAFs-exo promoted cell proliferation, migration, tube formation ability, and inhibited apoptosis of CRC cells. CAFs-sEV circN4BP2L2 knockdown reversed the above results. CircN4BP2L2 directly targeted miR-664b-3p, and HMGB3 was targeted by miR-664b-3p. Moreover, subcutaneous tumorigenesis and liver metastasis of nude mice with CRC were repressed by CAFs-sEV circN4BP2L2 knockdown. CAFs-sEV circN4BP2L2 knockdown restrained CRC cell proliferation and migration by regulating miR-664b-3p/HMGB3 pathway.

## Introduction

Colorectal cancer (CRC) has been regarded as one of the most common malignant tumors in gastrointestinal tract.^1^ Most patients are already in the advanced stage when diagnosed and have metastasis due to the insidious onset of CRC, inconspicuous early symptoms and low diagnosis rate, which increases the difficulty of clinical treatment.^2^ The occurrence of CRC is a complex multistep process.^3^ Cancer-associated fibroblasts cell (CAF) is an important component of tumor microenvironment, which regulates the recruitment and function of innate and adaptive immune cells in tumor microenvironment by secreting various growth factors, chemokines and proteases.^4,5^ It was reported that CAFs-derived exosomes could affect cell proliferation, migration and invasion of cancer cells.^6,7^ CAFs-derived exosomes enhanced drug resistance in CRC through priming cancer stem cells.^8^ LncRNAs, miRNAs and circular RNAs (circRNAs) participate in the crosstalks between CAFs and cancer cells, thereby accelerating tumor progression, which are enriched in exosomes.^4,9^ Some exosomal circRNAs have been shown to be candidated for potential diagnostic biomarkers and therapeutic targets of CRC.^10,11^ Uncovering the novel biomarkers and complicated mechanisms of CRC may have vital implication in improving therapeutic strategies and survival rate of CRC patients. •

As newly discovered endogenous RNAs, circRNAs form a closed loop structure through covalent bonds and regulate downstream gene expression via binding to targeted miRNAs, thus affecting multiple diseases including tumorigenesis.^12,13^ In recent years, a large number of studies have confirmed that CAFs can affect the progress of CRC by secreting a variety of RNAs or proteins, such as circSLC7A7, circPACRGL, etc.^14,15^ As demostrated by Xijuan Chen, CAF-derived exosomal miR-590-3p modulates the radioresistance in CRC.^16^ Similar to previous studies, our research group found that circN4BP2L2 was enriched in CAFs derived sEVs. However, what biological function of this phenomenon on the occurrence and development of CRC has not been reported yet. To explore the biological function of exosomal circN4BP2L2 secreted by CAFs in the occurrence and development of CRC will help us to find potential biomarkers and therapeutic targets for CRC. Hsa_circ_0006401 is generated from its host gene col6a3, which promotes cell proliferation and metastasis of CRC through regulating COL6A3 protein expression and its downstream TGFβ1 pathway.^17^ CircN4BP2L2 might be involved in the pathogenesis of epithelial ovarian cancer (EOC), and can be served as a diagnostic biomarker in patients with EOC.^18^ In our previous study, it was found that circN4BP2L2 (gene ID: hsa_circ_0005723) could promote CRC process via miR-340-5p/CXCR4 pathway.^19^ The miR-664b-3p was involved in cell progression of HCC.^20,21^ Starbase prediction showed that the expression of miR-664b-3p was significantly down-regulated in colon adenocarcinoma. Previous study reported that miR-664b-3p overexpression inhibited the proliferation and invasion of HCC cells.^20^ MiR-664b-3p could also serve as a tumor suppressor in the progression of colon cancer.^22^ However, the role of miR-664b-3p in CRC progression has not been investigated. It was predicted by the online website Starbase that circN4BP2L2 had targeted binding sites with miR-664b-3p, suggesting that circN4BP2L2 might serve as a competitive endogenous RNA (ceRNA).

High mobility group box 3 (HMGB3) belongs to the HMGB family, which promotes CRC process by regulating WNT/β-catenin pathway.^23^ As a cancer-promoting gene, HMGB3 can promote tumorigenesis and progression of CRC.^24^ Also, HMGB3 promotes the progression of glioma.^25^ Moreover, oncogenic signaling pathways such as Wnt/β-catenin axis could regulate cell proliferation, metastasis and angiogenesis of CRC.^23,26^ As proved by P. Han, et al., the activation of Wnt/β-catenin pathway was involved in CRC cell proliferation and chemoresistance.^27^ As predicted by bioinformation, miR-664b-3p has a targeted binding site with HMGB3, which has not been reported yet.

In the present study, the mechanism underlying the effect of CAFs-sEV circN4BP2L2 in CRC progression was presented. In general, our research indicated that CAFs-sEV circN4BP2L2 promoted CRC progression by regulating miR-664b-3p/HMGB3/Wnt/β-catenin pathway, which might provide a novel insight into the potential treatment target for CRC.

## Materials and methods

### Cell culture

The HUVECs and human CRC cell line LoVo were purchased from cell bank of Chinese Academy of Sciences (Shanghai, China). LoVo cells were cultured in DMEM medium (Gibco, USA) supplemented with 10% FBS, 100 mg/ml streptomycin and 100 U/ml penicillin (Invitrogen, CA, USA), and maintained in a humidified CO_2_ incubator at 37°C. HUVECs were cultured in ECGM medium (Thermo Fisher, USA) containing 10% FBS, and maintained at 37°C in 5% CO_2_ incubator.

### Isolation and culture of CAFs

CRC tissues and adjacent tissues were collected from Xiangya Hospital, Central South University. The informed consent forms were signed by all subjects. Our work was approved by the Medical Ethics Committee of Xiangya Hospital, Central South University. Human CAFs and NFs were isolated from CRC tissues and paracancerous tissues. After rinsing with sterile PBS buffer, all tissues were digested with collagenase I (Sigma, USA) at 37°C. The tissues were centrifuged and washed by DMEM medium, then cultured in DMEM medium with supplementation of 10% FBS, 1% penicillin and streptomycin (Invitrogen). The primary fibroblasts extracted from CRC tissues were named “CAFs”, and those from paracancerous tissues were named “NFs”. After passage 3 of culture, these fibroblasts were used for further research.

### Extraction and identifcation of sEVs

CAFs-sEV and NFs-sEV were extracted using total exosome isolation kits (Invitrogen, USA) through ultra-centrifugation as per the protocol. The primary CAFs and NFs cells that had just been isolated were first cultured in the medium containing 10% FBS. Then, the above cells were placed in the medium without sEVs for 24 h before sEVs were isolated. The supernatants were separated from cell medium and centrifuged at 2000 g for 30 min at 4°C, 10000 g for 40 min. It is then filtered through a filter (0.22 μm), and ultracentrifuged at 100000 g for 70 min to separate the supernatants and debrises. The debrises were rinsed with PBS buffer, centrifuged at 100000 g for 70 min, then resuspended in PBS buffer.

The electron microscopy was used to identify morphology of sEVs. The sEV suspensions were dripped onto a copper grid, and then they were negatively stained using 3% sodium phosphotungstate solution for 5 min. Subsequently, the sEVs were washed by H_2_O and dried. The transmission electron microscope (Philips, Netherlands) was used for sEVs detection.

The particle size of sEV was measured by NanoSight Tracking Analysis (NTA) and the levels of sEV markers CD9, CD63 and GM130 were determined using western blot assay with adding equal amounts of protein.

### Co-culturing of sEVs and CRC cells

Cells in the control group were cultured in PBS buffer without primary antibodies and CAFs-sEV and NFs-sEV were pre-labeled with PKH26 red fluorescent dye using a commercial kit (Sigma, USA). Then, the mixture was ultracentrifuged at 150,000 g for 1 h to remove the free dye with supplementation of PBS buffer plus 5% FBS. Co-culturing of PKH26-labeled sEVs and LoVo cells was conducted in a CO_2_ incubator at 37°C for 48 h. After co-incubation, they were fixed with 4% paraformaldehyde, and DAPI dye was used for nuclear staining (blue fluorescence). The laser scanning confocal microscope (LSM 510, Carl Zeiss, Germany) and TCS SP2 (Leica Microsystems, Germany) were applied for sEVs analysis.

### Immunofluorescent staining

The cells were fixed with 4% paraformaldehyde for 15 min, and then permeabilized using 0.5% Triton X-100. Subsequently, the cells were incubated with anti-α-smooth muscle actin (α-SMA, 1:200, Cell Signaling Technology, USA), anti-fibroblast activation protein (FAP-1, 1:200, Abcam, USA) or anti-fibroblast-specific protein-1 (FSP-1, 1:100, Abcam, USA) antibodies overnight at 4°C. Then, they were subjected to goat anti-rabbit IgG (1:2000, Abcam, USA) and incubated for 45 min. The cells were rinsed by PBS buffer for three times and cultured with 0.5 µg/ml DAPI. After rinsing by PBS buffer, the immunofuorescence was measured using a fluorescence microscope (Olympus, Japan).

### Cell transfection

The HMGB3 over-expressed vectors (Oe-HMGB3) were generated, sh-N4BP2L2, miR-664b-3p mimics and miR-664b-3p inhibitor, and the corresponding NCs were purchased from Shanghai GenePharma Company (China). Lipofectamine 2000 reagent (Invitrogen, USA) was utilized for cell transient transfection as per the protocol. Co-culturing of NFs-sEVs (40 μg/ml), CAFs-sEVs (40 μg/ml) and LoVo cells were conducted for 48 h. CAFs were transfected with sh-N4BP2L2 or sh-NC, and the corresponding sEVs were isolated and co-cultured with LoVo cells. CAFs-sEV/sh-N4BP2L2 and CAFs-sEV/sh-NC were added to the culture medium and co-cultured with miR-664b-3p inhibitor or inhibitor NC in LoVo cells. The control group was LoVo cells with PBS treatment. Meanwhile, the LoVo cells were also transfected with miR-664b-3p mimics or co-transfected with Oe-HMGB3 and miR-664b-3p mimics. NFs-sEVs, CAFs-sEVs and Lovo cells were co-cultured for 48 h, respectively, and the equal seeding densities of cells were taken (500 μL, 800 mg/mL) to detect the cell migration and angiogenesis abilities. The timelines of cell experiments were shown in the Figure S4A.

### CCK-8 assay

Cell proliferation was assessed by using CCK-8 kit (Dojindo, Japan). 5 × 10^3^ cells/mL of LoVo cells were inoculated into a 96-well plate. After mixing 10 μL of CCK-8 solution into each well at 0 h, 24 h, 48 h and 72 h, the cells were incubated for 2 h at 37°C. The optical density (OD) value was detected at 450 nm using a microplate reader (Bio-rad Laboratories, USA) at room temperature.

### Apoptosis detection using flow cytometry

Cell apoptosis was evaluated by flow cytometry using Annexin V-FITC/PI staining kit (Invitrogen, USA). After harvesting, the LoVo cells were trypsinized, rinsed by cold PBS buffer and stained with 5 µL Annexin V-FITC and 10 µL PI solution. Subsequently, the cell apoptosis rate was quantitatively assessed using FACScan flow cytometer (Beckman Coulter, USA).

### Transwell assay

Transwell assay was carried out for cell migration detection. The upper chamber was filled with 0.5 mL of serum-free medium, followed by inoculating of cells into the upper chamber of the transwell. Meanwhile, 600 µL of DMEM medium containing 10% FBS was mixed into the lower chamber. Cells were incubated in an incubator for 24 h at 37°C. Subsequently, the cells were fixed with 4% paraformaldehyde and stained with crystal violet (Sigma, USA). The cells were visualized and calculated by a microscope.

### Tube formation

LoVo cells (1 × 10^5^/well) were cultured until 70% confluence, then serum-free DMEM culture medium instead of the complete DMEM medium was used for cell culture. After culture for 48 h, the conditional medium was centrifuged. Subsequently, 1 × 10^5^/well of HUVECs were suspended in conditional medium and inoculated into 24-well cell culture plate coated with 300 μL of Matrigel matrix (BD Biosciences, USA). After incubating at 37°C for 24 h, five fields were randomly selected from each well to observe and record the formed capillary-like structures under a microscopy to monitor the tube formation ability of HUVECs.

### Dual-luciferase reporter assay

The targeted binding sites between circN4BP2L2 and miR-664b-3p, as well as miR-664b-3p and HMGB3 were predicted by Starbase online software. The wild-type and mutant-type sequences of CircN4BP2L2 3’-UTR (CircN4BP2L2-WT/CircN4BP2L2-MUT) containing the predicted binding site were cloned into pmirGLO vector (GenePharma, China). CircN4BP2L2-WT or CircN4BP2L2-MUT and miR-642a-3p mimics, miR-513c-5p mimics, miR-526b-5p mimics, miR-519a-3p mimics and miR-664b-3p mimics were co-transfected by the Lipofectamine 3000 reagent (Invitrogen, USA). After transfection for 24 h, a dual-luciferase reporter assay system (Promega, USA) was applied to monitor the luciferase activity. Besides, the target relationship between miR-664b-3p and HMGB3 was verified using the same method.

### Mice xenograft assay and liver metastasis

A total of 36 BALB/c male nude mice, aged 5 to 6 weeks old, weighting (18 ± 2) g, provided by Animal Center of the Chinese Academy of Science (Beijing, China) were housed under controlled laboratory conditions. All animal experiments were approved by the Ethics Committee of Xiangya Hospital, Central South University. The mice were randomly divided into control group, CAFs-sEV/sh-NC group and CAFs-sEV/sh-N4BP2L2 group, with six mice in each group. The LoVo cells (5.0 × 10^6^ cells/mouse, 100 μL) were treated with CAFs-sEV/sh-NC (50 μg/mL) or CAFs-sEV/sh-N4BP2L2 (50 μg/mL) and injected subcutaneously into the dorsal sides of mice. In the mice xenograft assay experiment, sEVs were injected into mice on day 1 and day 4, respectively. The control group of Mice xenograft assay was injected with equal amounts of LoVo cells. The tumor length and width were monitored every 7 days and the volume of tumor was calculated according to (length × width^2^)/2. After euthanizing on the 35th day, the tumor tissues of the mice were excised and weighed. Then the tissues were fixed with 10% formaldehyde. The paraffin sections were routinely made and rabbit anti-Ki67 antibody (Cell Signaling Technology, USA) was used for immunohistochemistry assay.

For detection of liver metastasis, the mice were injected with 1 × 10^6^ cells with CAFs-sEV/sh-NC (50 μg/mL) or CAFs-sEV/sh-N4BP2L2 (50 μg/mL) treatment by tail vein. In the liver metastasis experiment, the mice were injected twice a week until the end of the experiment. After 42 days injection, the mice were sacrificed and liver metastasis of CRC cells was evaluated. The liver tissues were imaged and the numbers of metastatic nodules liver were counted. Meanwhile, the liver tissues were fixed with formaldehyde, embedded in paraffin, and stained with hematoxylin and eosin (HE) (Solarbio, China). The timelines of animal experiments were shown in Fig. S4B.

### Western blot assay

Total proteins were extracted with the steps of the instructions of RIPA reagent (Beyotime, China); then, the protein concentrations were measured. The proteins were separated by SDS-PAGE, and electrically transferred to the PVDF membranes (Invitrogen, USA). The samples (30 μg for each sample) were blocked in 5% nonfat milk for 60 min. Subsequently, primary antibodies against α-SMA, FAP-1, FSP-1, CD9, CD63, p-GSK-3β, GSK-3β, HMGB3, GAPDH at 1:1000 dilution and β-catenin at 1:5000 dilution, all purchased from Abcam, USA, were incubated overnight at 4°C. Then the goat horseradish peroxidase-conjugated anti-rabbit IgG was applied. The electrochemical luminescence detection system was used to visualize immunoreactive bands as per the instructions. Each protein level is normalized to GAPDH level.

### Quantitative real-time polymerase chain reaction (qRT-PCR)

Total RNA was extracted using TRIzol reagent (Invitrogen, USA) and RNA concentration was determined. Reverse transcription was conducted by using reverse transcription kit (Takara, China) according to the instructions. The qRT-PCR assay was carried out to measure the relative expression levels of mRNA using SYBR Green Master PCR mix (Applied Biosystems) under the guidance of manufacturer’s protocol. GAPDH and U6 were served as the internal reference and the relative expression of gene was calculated by 2^−ΔΔCt^ method. The primers used in this work were as follows:

circN4BP2L2 (F): 5’- CAAAGACCTCCTCCTCCACA −3’;

circN4BP2L2 (R): 5’- TCAGTGCTGAACACAATGCC −3’;

miR-664b-3p(F): 5’- CGTCCTTCATTTGCCTCCCAG −3’;

miR-664b-3p (R): 5’- GCAGGGTCCGAGGTATTC −3’;

HMGB3(F): 5’- AGATGGCCACAGTAGCAAGT-3’;

HMGB3(R): 5’- CCTGCGTGTTTCATAGCCTC-3’;

U6 (F): 5’- CTCGCTTCGGCAGCACA-3’;

U6 (R): 5’- AACGCTTCACGAATTTGCGT-3’;

GAPDH (F): 5’- CCAGGTGGTCTCCTCTGA-3’;

GAPDH (R): 5’- GCTGTAGCCAAATCGTTGT-3’.

### Statistical analysis

The tests conducted in this study were repeated at least for three times. SPSS22.0 software package (SPSS Inc., USA) was used for statistical data analysis in this study. The values were shown as means ± standards deviation. The pairwise comparison was performed using student’s t test and the one-way ANOVA was used for multi-group contrast. Statistically significant differences were defined as *P* < .05.

## Results

### Identification of CAFs cells and the sEV characterization

**Figure 1.**
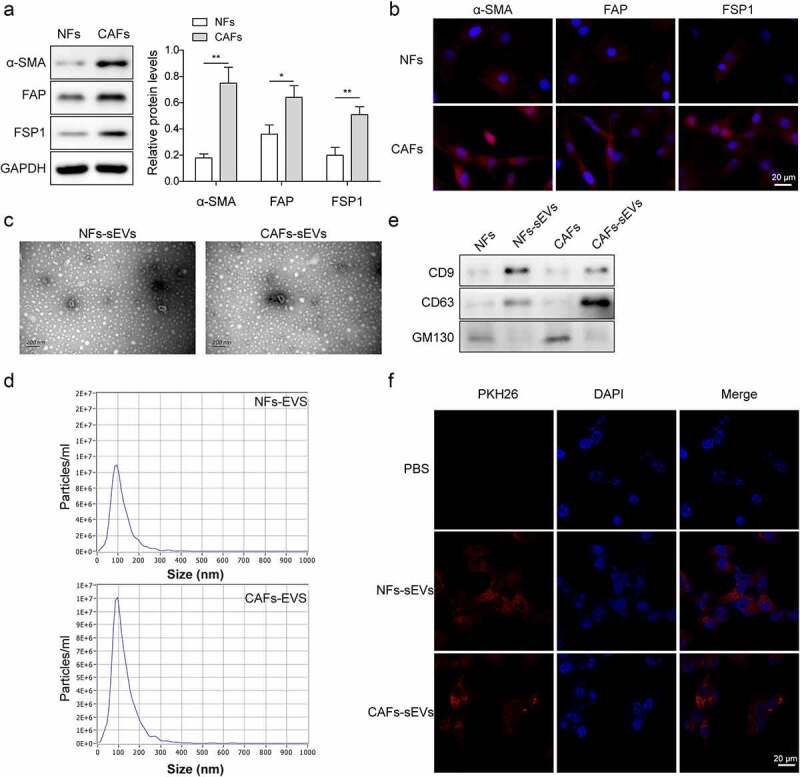
Identification of CAFs cells and the characterization of sEV.

CRC tissues and paracancerous normal tissues were used for human CAFs and NFs isolation. Western blot and immunofluorescent staining results indicated that activated fibroblast-specific markers (α-SMA, FAP, FSP1) were expressed in both CAFs and NFs cells, but these proteins were higher in CAFs than NFs (Figure 1a,b). To investigate the effects of circRNA of CAFs-sEVs on CRC, sEVs were extracted by differential centrifugation. The morphology of sEV and the particle size were analyzed by electron microscopy and NanoSight Tracking Analysis (NTA), respectively (Figure 1c,d). The median size of NFs-sEVs and CAFs-sEVs was 100 nm. Besides, the sEV markers (CD9 and CD63) were positively expressed in CAFs-sEV and NFs-sEV, while GM130 was negatively expressed (Figure 1e). The above data revealed that sEVs were successfully extracted from CAFs and NFs. In addition, after co-culturing of PKH26-labeled CAFs-sEV and NFs-sEV, the sEV uptake by LoVo cells was observed using laser confocal microscopy (figure 1f).

(a) The levels of activated fibroblast-specific markers α-SMA, FAP, FSP1 in both CAFs and NFs cells were detected by western blot. Each protein level is normalized to GAPDH level. (b) Immunofluorescent staining was conducted to detect the positive rates of α-SMA, FAP and FSP1 proteins in both CAFs and NFs cells. Blue fluorescence represents DAPI staining of cell nucleus, and red fluorescence is KI67 protein (magnification: 200×, scale bar: 20 μm). (c-d) The morphology of sEV was identified using electron microscopy and the particle size was measured by NTA (scale bar: 200 nm). (e) Expressions of CD9, CD63 and GM130 were measured using western blot. (f) sEV uptake of LoVo cells was observed by laser confocal microscopy. The red fluorescence represents Ki67 and blue fluorescence represents DAPI staining of cell nucleus (magnification: 200×, scale bar: 20 μm). *P < .05, **P < .01. *Comparison with NFs group. Each experiment in this work was conducted independently at least three times.

### Knockdown of CAFs-derived sEV circN4BP2L2 restrained proliferation and migration of CRC cells

**Figure 2.**
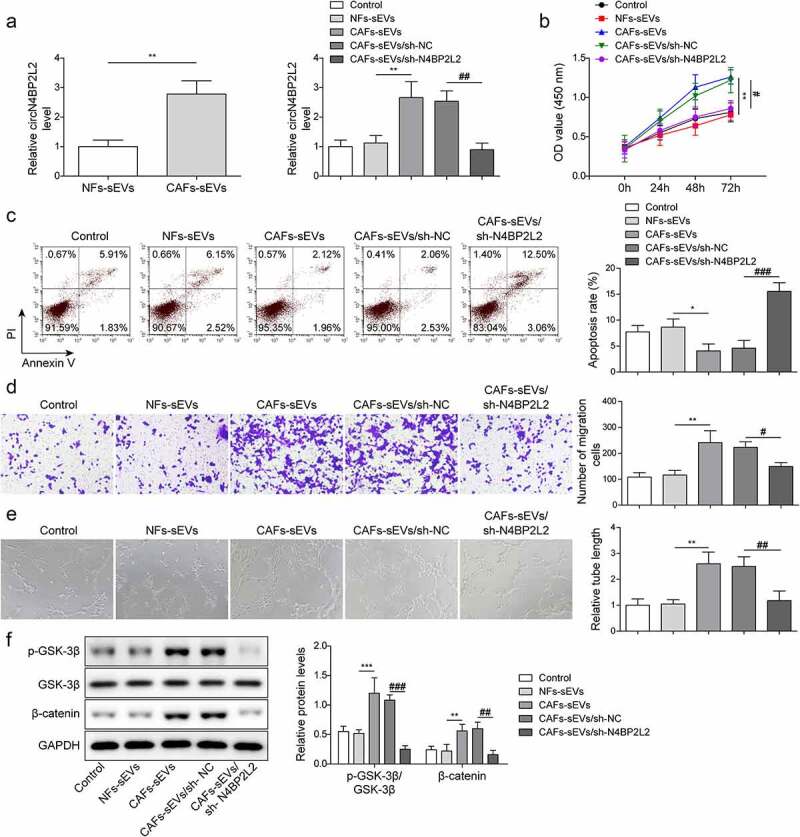
CircN4BP2L2 knockdown inhibits CRC progression.

As shown in Figure 2a, the expression of circN4BP2L2 was elevated in CAFs-sEV and LoVo cells. To further explore the function of circN4BP2L2 in CRC, we knocked out the expression of circN4BP2L2 in CAFs, and co-cultured the extracted sEVs with LoVo cells. CircN4BP2L2 expression in CAFs cells after transfection of sh-NC and sh-circN4BP2L2 was detected by qRT-PCR. The results showed that the expression of circN4BP2L2 was significantly decreased after transfection of sh-circN4BP2L2 (Figure S1A). The cell viability of LoVo cells was increased after CAFs-sEV treatment, while it was decreased after CAFs-sEV/sh-N4BP2L2 treatment (Figure 2b). On the contrary, the apoptosis rate of LoVo cells was declined with CAFs-sEV addition, while it was elevated after the addition of CAFs-sEV/sh-N4BP2L2 (Figure 2c). CAFs-sEV group showed strengthened migration capability in LoVo cells, whereas CAFs-sEV/sh-N4BP2L2 group behaved in weakened cell migration ability (Figure 2d). The tube formation ability of CAFs-sEV group was promoted, while it was repressed in CAFs-sEV/sh-N4BP2L2 group (Figure 2e). Besides, GSK-3β and β-catenin phosphorylation levels were elevated in LoVo cells incubated with CAFs-sEV, which led to the activation of Wnt/β-catenin pathway, while the above results were reversed in CAFs-sEV/sh-N4BP2L2 group (figure 2f). Furthermore, NFs-sEVs, CAFs-sEVs and Lovo cells were co-cultured for 48 h, respectively, and the equal seeding densities of cells were taken (500 μL, 800 mg/mL) to detect the cell migration and angiogenesis abilities. The results revealed that the cell migration and angiogenesis abilities were still weakened after CAFs-sEVs treatment (Fig. S2A-B). These data proved that silencing of CAFs-sEV circN4BP2L2 inhibited CRC cell progression and Wnt/β-catenin signal pathway.

(a) The expressions of circN4BP2L2 with NFs-sEV or CAFs-sEV treatment were assessed by qRT-PCR assay (left panel). The expressions of circN4BP2L2 in CAFs-sEV and CAFs-sEV/sh-N4BP2L2 groups were determined by qRT-PCR assay (right panel). The “Control” was human CRC cell line LoVo with PBS treatment. (b) Cell proliferation ability of LoVo cells in CAFs-sEV and CAFs-sEV/sh-N4BP2L2 groups was measured using CCK-8 method. (c) Flow cytometry was conducted to measure the apoptosis rate of LoVo cells in CAFs-sEV and CAFs-sEV/sh-N4BP2L2 groups. (d) Transwell assay was carried out to indicate cell migration ability in CAFs-sEV and CAFs-sEV/sh-N4BP2L2 groups. (e) Tube formation ability at 0 h and 24 h was determined in HUVECs cells in CAFs-sEV and CAFs-sEV/sh-N4BP2L2 groups. (f) The phosphorylation level of GSK-3β and β-catenin expression was determined by western blot assay in CAFs-sEV and CAFs-sEV/sh-N4BP2L2 groups. Each protein level is normalized to GAPDH level. *P < .05, **P < .01, ***P < .001; #*P* < .05, ##*P* < .01, ###*P* < .001. *Comparison with NFs-sEV group; # comparison with CAFs-sEVs/sh-NC group. Each experiment in this work was conducted independently at least three times.

### CircN4BP2L2 directly targeted miR-664b-3p and negatively regulated its expression

**Figure 3.**
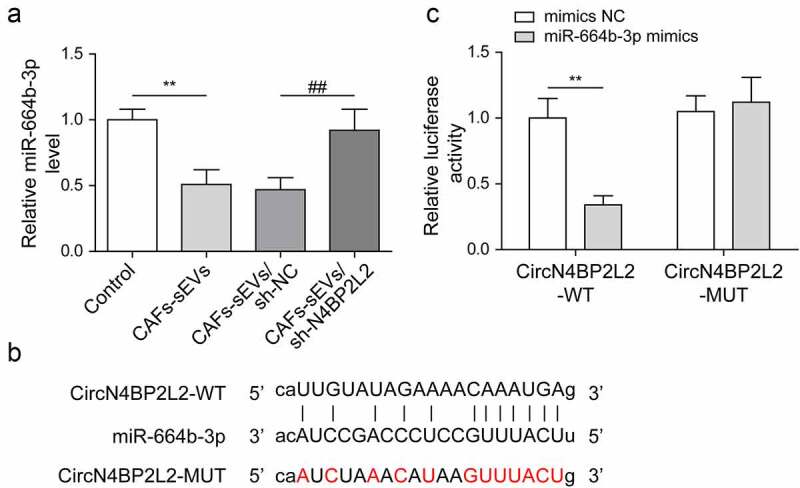
CircN4BP2L2 directly targets miR-664b-3p and negatively regulates its expression.

After co-culturing of CAFs-sEV and LoVo cells, miR-664b-3p expression was down-regulated, while the expression of miR-664b-3p was up-regulated in LoVo cells with CAFs-sEV/sh-N4BP2L2 treatment (Figure 3a). For exploring the relationship between circN4BP2L2 and miR-664b-3p in CRC, Starbase prediction was used and found that circN4BP2L2 and miR-664b-3p had binding sites (Figure 3b). Subsequently, the luciferase reporter assay further verified that circN4BP2L2 targeted regulated miR-664b-3p expression (Figure 3c). Starbase was used to predict the targeted miRNAs of circN4BP2L2, and Starbase, TargetScan and miRWalk were used to predict the targeted miRNAs of HMGB3. The intersection of miRNAs was obtained and there were 45 miRNAs were found, including miR-664b-3p (Fig. S3A). MiR-642a-3p, miR-513c-5p, miR-526b-5p, miR-519a-3p and miR-664b-3p were screened out. Subsequently, the luciferase reporter assay result revealed that miR-664b-3p mimics significantly down-regulated the activity of circN4BP2L2-WT, while there was no statistic difference of circN4BP2L2-MUT between mimic NC and miR-664b-3p mimics (Fig. S3B). Moreover, starbase prediction showed that the expression of miR-664b-3p was significantly down-regulated in colon adenocarcinoma (Fig. S3C). These data suggested that circN4BP2L2 had directly binding sites with miR-664b-3p and negatively regulated miR-664b-3p expression in CRC cells.

(a) qRT-PCR assay was carried out to check the level of miR-664b-3p in LoVo cells with sh-N4BP2L2 transfection. The “Control” was human CRC cell line LoVo with PBS treatment. (b) The prediction of binding site between circN4BP2L2 and miR-664b-3p was conducted using Starbase online software. (c) The targeted relationship between circN4BP2L2 and miR-664b-3p was verified by dual-luciferase reporter assay. *P < .05, **P < .01, ***P < .001; #*P* < .05, ##*P* < .01, ###*P* < .001. * Comparison with the control group; # comparison with the CAFs-sEVs/sh-NC group. Each experiment in this work was conducted independently at least three times.

### Repression of miR-664b-3p weakened the effects of circN4BP2L2 knockdown on proliferation and migration of CRC cells

**Figure 4.**
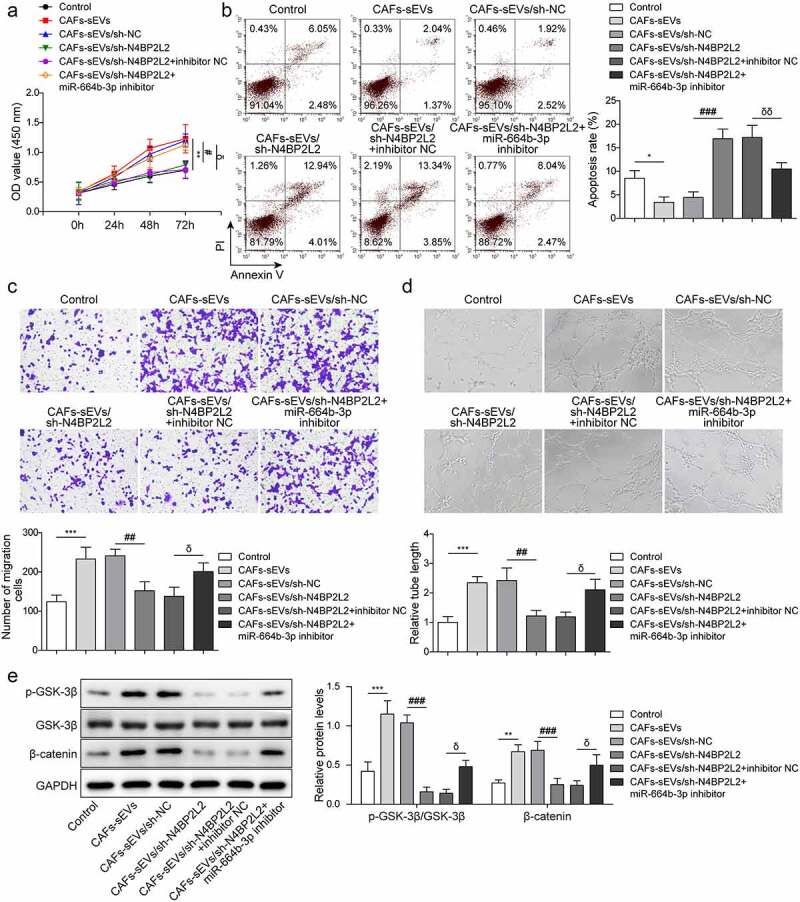
Repression of miR-664b-3p weakened the effects of circN4BP2L2 knockdown on proliferation and migration of CRC cells.

To further investigate the cross-talk between circN4BP2L2 and miR-664b-3p in CRC cell progression, CAFs-sEV/sh-N4BP2L2 was co-transfected with miR-664b-3p inhibitor or inhibitor NC in LoVo cells. As demonstrated by CCK-8 assay, knockdown of miR-664b-3p resulted in increased cell viability of LoVo cells, which attenuated CAFs-sEV/sh-N4BP2L2-induced decline of cell viability (Figure 4a). After miR-664b-3p knockdown, the apoptosis rate of LoVo cells lowered, which reversed the results of CAFs-sEV/sh-N4BP2L2 treatment (Figure 4b). The restraint effect of cell migration ability caused by CAFs-sEV/sh-N4BP2L2 treatment was reversed due to miR-664b-3p inhibitor co-transfection (Figure 4c). Besides, CAFs-sEV/sh-N4BP2L2 treatment decreased tube formation amount of LoVo cells, and the result was reversed after miR-664b-3p (Figure 4d). Moreover, the Wnt/β-catenin pathway was activated after miR-664b-3p knockdown, reversing CAFs-sEV/sh-N4BP2L2 treatment caused inhibition of this pathway (Figure 4e). Based on these results, CAFs-sEV circN4BP2L2 participates in CRC progression by sponging miR-664b-3p.

CAFs-sEV/sh-N4BP2L2 was co-transfected with inhibitor NC or miR-664b-3p inhibitor in LoVo cells. (a) CCK-8 assay was conducted to evaluate cell proliferation ability. (b) Cell apoptosis rate was evaluated using flow cytometry. (c) The transwell assay was performed to assess cell migration ability. (d) The tube formation ability at 0 h and 24 h was determined in HUVECs cells. (e) Western blot was conducted to determine GSK-3β and β-catenin levels. Each protein level is normalized to GAPDH level. *P < .05, **P < .01, ***P < .001; #*P* < .05, ##*P* < .01, ###*P* < .001; δ*P* < .05, δδ*P* < .01, δδδ*P* < .001. * Comparison with control group; # comparison with CAFs-sEVs/sh-NC group; δcomparison with CAFs-sEV/sh-N4BP2L2 + inhibitor NC group. The control group in Figure 4 was LoVo cells with PBS treatment. Each experiment in this work was conducted independently at least three times.

### MiR-664b-3p negatively regulated HMGB3 expression

**Figure 5.**
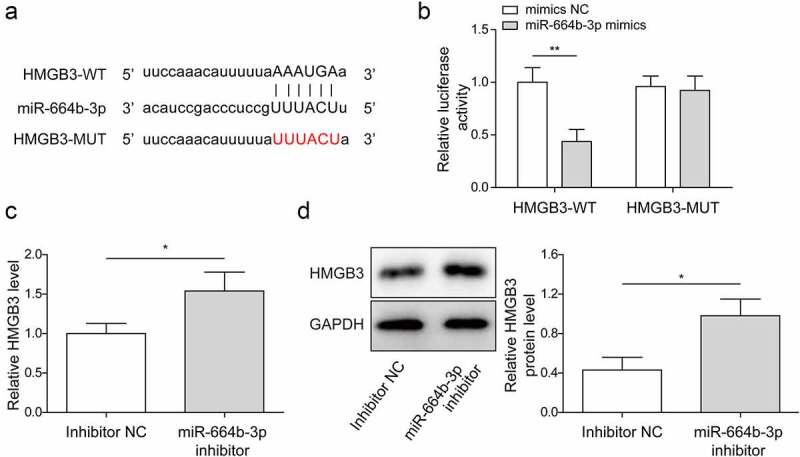
MiR-664b-3p negatively regulated HMGB3 expression.

HMGB3 has been proved to promote the occurrence and progression of CRC.^24^ Searching from online software Starbase, HMGB3 had binding sites with miR-664b-3p (Figure 5a). Dual-luciferase reporter gene detection disclosed that luciferase activity was reduced in LoVo cells with HMGB3-WT and miR-664b-3p mimics co-transfection, while it was not affected in HMGB3-MUT cells (Figure 5b). Besides, the mRNA and protein levels of HMGB3 were up-regulated in LoVo cells with miR-664b-3p inhibitor transfection (Figure 5c,d). The above data provided that HMGB3 was a potential target of miR-664b-3p.

(a) The potential binding site between HMGB3 and miR-664b-3p was illustrated by bioinformatics tool Starbase. (b) The targeted relationship of miR-664b-3p and HMGB3 was verified by dual-luciferase reporter assay. *P < .05, **P < .01, ***P < .001. * Comparison with mimics NC group. (c-d) HMGB3 mRNA and protein expressions were detected using qRT-PCR and western blot. *P < .05, **P < .01, ***P < .001. * Comparison with the inhibitor NC group. Each protein level is normalized to GAPDH level. *P < .05, **P < .01, ***P < .001. Each experiment in this work was conducted independently at least three times.

### HMGB3 overexpression eliminated the effects of miR-664b-3p overexpression on CRC cell progression

**Figure 6.**
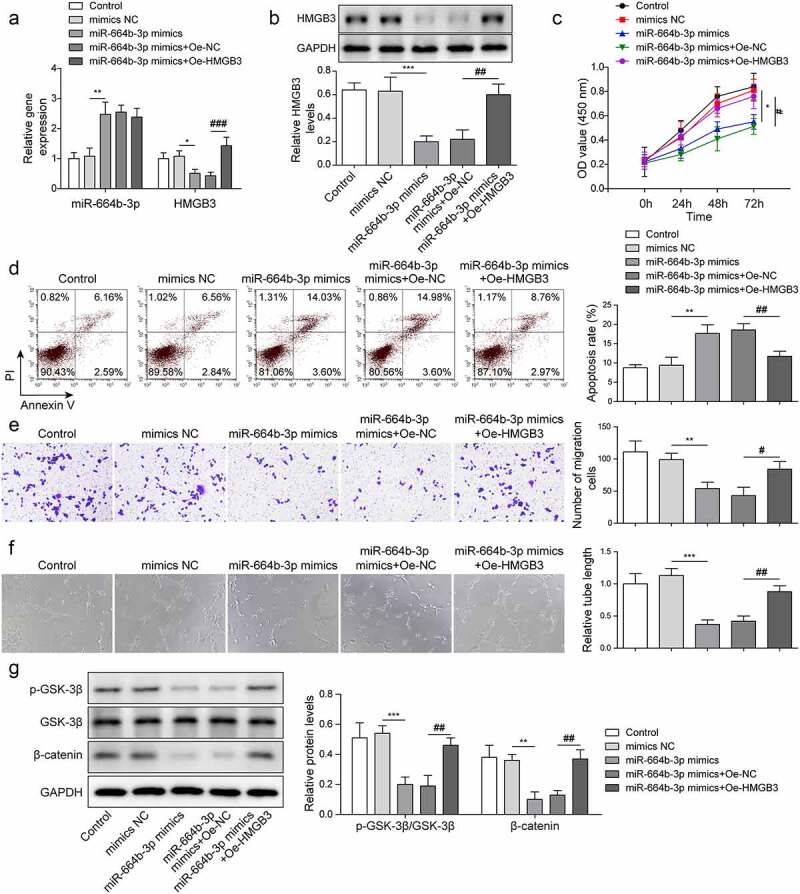
HMGB3 overexpression eliminated the effects of miR-664b-3p overexpression on CRC cell progression.

In the next step, we sought to investigate whether miR-664b-3p functioned in CRC by inhibiting HMGB3 expression. As shown in Figure 6a –b, HMGB3 expression in LoVo cells was downregulated after overexpression miR-664b-3p, while it was reversed after HMGB3 was over-expressed. However, the expression of miR-664b-3p was not changed after HMGB3 overexpression. In order to explore the efficiency of HMGB3 overexpression, we used western blot assay to detect HMGB3 protein expression in LoVo cells after transfection of Oe-NC and Oe-HMGB3 (Fig. S1B). The results showed that the expression of HMGB3 protein was significantly increased after transfection of Oe-HMGB3, indicating the success of HMGB3 over-expression construct. With miR-664b-3p mimics transfection, the cell proliferation was drastically depleted in LoVo cells, while it was elevated after miR-664b-3p mimics and Oe-HMGB3 co-transfection (Figure 6c). On the contrary, the cell apoptosis rate was elevated with miR-664b-3p mimics transfection, and the effect was restrained by the simultaneous Oe-HMGB3 transfection (Figure 6d). Additionally, miR-664b-3p mimics weakened cell migration and tube formation abilities, while synchronous HMGB3 over-expression markedly reversed the above effects (Figure 6e,f). Furthermore, after miR-664b-3p mimics transfection, Wnt/β-catenin pathway was suppressed, while the effect was reversed by Oe-HMGB3 reintroduction (Figure 6g). These findings illustrated that HMGB3 overexpression eliminated the effects of miR-664b-3p overexpression on CRC cell progression.

LoVo cells were transfected with mimics NC, miR-664b-3p mimics, miR-664b-3p mimics + Oe-NC and miR-664b-3p mimics + Oe-HMGB3. (a-b) MiR-664b-3p and HMGB3 levels in LoVo cells were measured using qRT-PCR and western blot. Each protein level is normalized to GAPDH level. (c) The cell proliferation was estimated by CCK-8 method. (d) Flow cytometry was conducted to measure the apoptosis rate. (e) Cell migration ability was monitored by transwell assay. (f) Tube formation ability at 0 h and 24 h was determined in HUVECs cells. (g) The phosphorylation level of GSK-3β and β-catenin was measured using western blot. The control group in Figure 6 was LoVo cells with PBS treatment. *P < .05, **P < .01, ***P < .001; #*P* < .05, ##*P* < .01, ###*P* < .001. * Comparison with mimics NC group; # comparison with miR-664b-3p mimics + Oe-NC group. Each experiment in this work was conducted independently at least three times.

### Knockdown of CAFs-derived sEV circN4BP2L2 inhibited subcutaneous tumorigenesis and liver metastasis in nude mice with CRC

**Figure 7.**
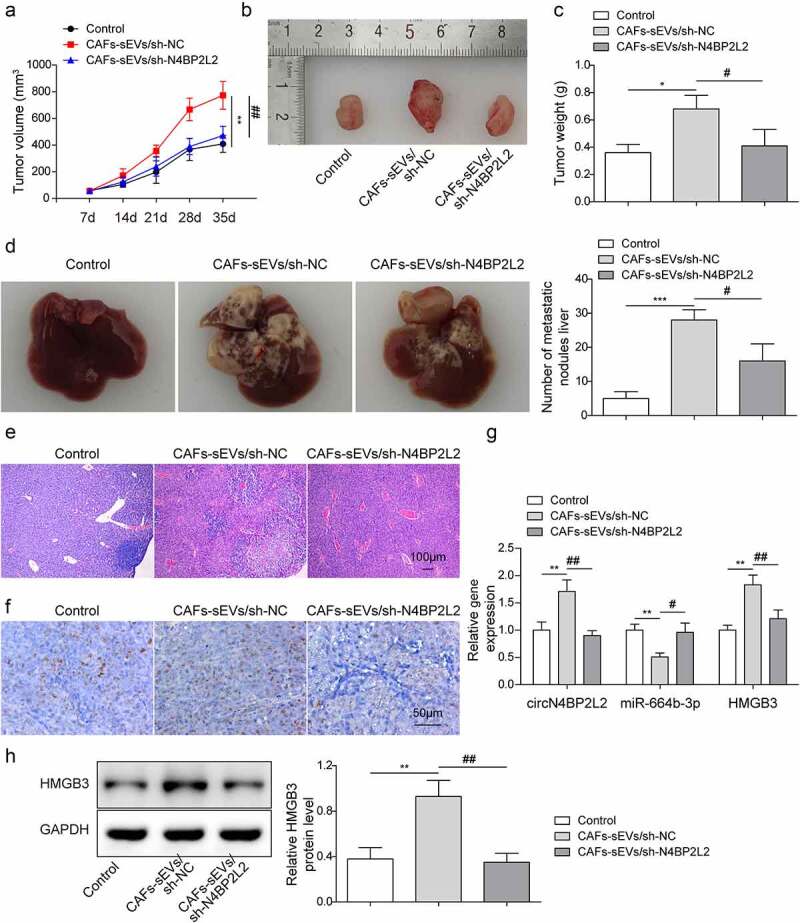
Knockdown of CAFss-derived sEV circN4BP2L2 inhibited subcutaneous tumorigenesis and liver metastasis of nude mice with CRC.

To explore the possible regulation of circN4BP2L2 *in vivo*, the LoVo cells with CAFs-sEV/sh-NC or CAFs-sEV/sh-N4BP2L2 treatment were subcutaneously injected into the nude mice. As shown in Figure 7a-c, CAFs-sEV/sh-NC group had strikingly increased tumor volume and weight compared with those in the control group, while the values were decreased in the CAFs-sEV/sh-N4BP2L2 group. Furthermore, liver metastasis and HE staining showed that after the injection of CAFs-sEV, the liver metastasis was increased, while after the knockdown of circN4BP2L2, the liver metastasis was dramatically decreased (Figure 7d,e). Immunohistochemistry assay indicated that Ki67 levels was elevated in tumor tissues after injection of CAFs-sEV, while the expression of Ki67 was notably depleted after circN4BP2L2 knockdown (figure 7f). Moreover, after the injection of CAFs-sEV, the expressions of circN4BP2L2 and HMGB3 in tumor tissues were enhanced, while miR-664b-3p expression was reduced, which was reversed after circN4BP2L2 knockdown (Figure 7g,h). The above results displayed that CAFs-sEV circN4BP2L2 silencing restrained subcutaneous tumorigenesis and liver metastasis in CRC nude mice.

The LoVo cells with CAFs-sEV/sh-NC or CAFs-sEV/sh-N4BP2L2 treatment were subcutaneously injected into the nude mice. (a-c) The subcutaneous tumor volume, figure and weight in each group of nude mice were measrued in CAFs-sEV/sh-NC and CAFs-sEV/sh-N4BP2L2 groups. (d) The figures of liver tissue morphology were photoed and the number of liver metastases was counted. (e) HE staining was conducted to detect liver metastases. (f) Immunohistochemistry assay was conducted to indicate Ki67 expression. (g-h) The circN4BP2L2 and HMGB3 expressions in tumor tissues were examined by qRT-PCR and western blot. The control group of Figure 7 was injected with equal amounts of LoVo cells. Each protein level is normalized to GAPDH level. n = 6. *P < .05, **P < .01, ***P < .001; #*P* < .05, ##*P* < .01, ###*P* < .001. * Comparison with control group; # comparison with CAFs-sEVs/sh-NC group.

## Discussion

CRC is one of the most common malignancies worldwide.^28,29^ The molecular regulation mechanism of CRC is complex and the exploration of specific biomarkers is helpful for clinical diagnosis and treatment of CRC. CAFs could secret sEVs to CRC cells, thereby promoting the development and metastasis of CRC and participating in the therapeutic resistance of CRC cells.^28,30^ In this study, we reported that CAFs-derived sEV circN4BP2L2 accelerated CRC progression via regulating miR-664b-3p/HMGB3 expression, as well as activating the Wnt/β-catenin pathway.

CAFs-exo miR-148b-3p was involved in the progression of bladder cancer progression through via regulating Wnt/β-catenin pathway.^31^ CAF-derived exosomes transported LINC00659 in CRC cells, which promoted cell proliferation by acted serving as a ceRNA sponge for miR-342-3p to promote cell proliferation.^4^ The circN4BP2L2 played an oncogenic role in CRC by sponging miR-340-5p to competitively enhance CXCR4 expression.^19^ Moreover, circN4BP2L2 was related to a variety of clinicopathological features of EOC, which might serve as a novel prognostic biomarker in patients with EOC.^18^ Meanwhile, circN4BP2L2 could act as an adjunct to Cancer antigen 125 (CA125) and human epididymis protein 4 (HE4) for EOC detection, especially in early stage of EOC.^32^ It was found in this study that CAFs-sEV circN4BP2L2 knockdown restrained cell proliferation, migration and tube formation ability of CRC cells, repressed Wnt/β-catenin signal pathway, and promoted apoptosis (Figure 2). Cell growth states, such as cell proliferation and apoptosis, will affect other functions of cells, such as migration, invasion and angiogenesis. However, the effects of cell proliferation and apoptosis on cell migration and angiogenesis cannot be completely excluded at present. In this work, it was found that in CAFs-sEVs treated Lovo cells, cell migration and angiogenesis abilities remained impaired (Fig. S2). Besides, in vivo experiments showed that circN4BP2L2 silencing in CAFs repressed subcutaneous tumorigenesis and liver metastasis of nude mice with CRC (Figure 7). These results concluded that CAFs-sEV circN4BP2L2 might be responsible for CRC progression.

CircRNAs, as endogenous RNA molecules for miRNA sponges, regulate multiple biological processes by directly binding to targeted miRNAs.^33,34^ For example, circFMN2 promoted CRC cell proliferation via regulating the miR-1182/hTERT axis.^35^ As demonstrated by Shang A, et al., CRC-derived exosome circPACRGL could facilitate the progression of CRC cells through regulating the miR-142-3p/miR-506-3p-TGF-β1 pathway.^15^ In our work, starbase was used to predict the targeted miRNAs of circN4BP2L2, and Starbase, TargetScan and miRWalk were used to predict the targeted miRNAs of HMGB3. The intersection of miRNAs was obtained and there were 45 miRNAs were found, including miR-664b-3p (Fig. S3A). Then, we screened miRNAs that have not been reported in CRC through PubMed, and miR-642a-3p, miR-513c-5p, miR-526b-5p, miR-519a-3p and miR-664b-3p were screened out. Subsequently, the luciferase reporter assay result revealed that miR-664b-3p mimics significantly down-regulated the activity of circN4BP2L2-WT (Fig. S3B). Moreover, starbase prediction showed that the expression of miR-664b-3p was significantly down-regulated in colon adenocarcinoma (Fig. S3C). However, there was currently no literature report on the study of miR-664b-3p in CRC. MiR-664b-3p overexpression suppressed cell proliferation, migration, and invasion in colon cancer through restraining BUB3 protein expression.^22^ It was found here that miR-664b-3p overexpression drastically depleted cell proliferation, migration and tube formation abilities in LoVo cells and elevated cell apoptosis rate (Figure 6). Moreover, Dual-luciferase reporter assay verified that circN4BP2L2 could directly bind and negatively regulate miR-664b-3p expression (Figure 3). The effects caused by CAFs-sEV circN4BP2L2 knockdown were reversed after miR-664b-3p was repressed (Figure 4). These findings first imply the regulatory correlation betweencircN4BP2L2 and miR-664b-3p in CRC cell progression.

HMGB3 is involved in the progression of some cancers, such as esophageal squamous cell cancer, breast cancer and so on.^36,37^ HMGB3 has been demonstrated to play an oncogene role in CRC.^38^ miR-93 targeted regulated HMGB3 expression, and the repression effect of miR-93 on biological behavior of CRC cells could be counteracted by HMGB3 overexpression.^39^ In the current work, we proved that miR-664b-3p could directly target and negatively regulate HMGB3 expression (Figure 5). Meanwhile, miR-664b-3p enrichment weakened cell proliferation, cell migration and tube formation abilities, accelerated cell apoptosis in CRC cells, and the above effects were reversed due to HMGB3 over-expression (Figure 6). Thus, miR-664b-3p might serve as a tumor suppressor in the progression of CRC by restraining HMGB3 expression. Further studies are required to verify whether miR-664b-3p/HMGB3 pathway is associated with patient prognosis. In our work, circN4BP2L2-modulated signaling pathway was responsible for regulating CRC progression, providing a new insight into the potential biomarker and treatment target for CRC and it was suggested that CAFs-sEV circN4BP2L2 deserved further consideration to screen and diagnose CRC. However, due to the complicated components of exosome, whether CAFs-sEV circN4BP2L2 has other roles in regulating CRC progression remains to be further studied.

We reported that CAFs-sEV circN4BP2L2 exerted an oncogene role and promoted cell proliferation, migration and tube formation abilities by up-regulating HMGB3 expression via recruiting miR-664b-3p, which might provide a novel insight for the mechanism of circN4BP2L2 in CRC development.

## Supplementary Material

Supplemental Material

## Abbreviation

CRC: Colorectal cancer
CAFs: Cancer-associated fibroblasts
CAFs-exo: CAFs-derived sEV
NFs-exo: NFs-derived sEV
TME: Tumor microenvironment
CircRNAs: Circular RNAs
EOC: Epithelial ovarian cancer
CeRNA: Competitive endogenous RNA
HMGB3: High mobility group box 3
HUVECs: Human umbilical vein endothelial cells
FBS: Fetal bovine serum
NTA: NanoSight Tracking Analysis
