# Supplementary figures and images for: CAFs-derived small extracellular vesicles circN4BP2L2 promotes proliferation and metastasis of colorectal cancer via miR-664b-3p/HMGB3 pathway
Source: Cancer Biol Ther. 2022 Jun 19;23(1):404–16. doi: 10.1080/15384047.2022.2072164 (PMC9225373; doi:10.1080/15384047.2022.2072164)

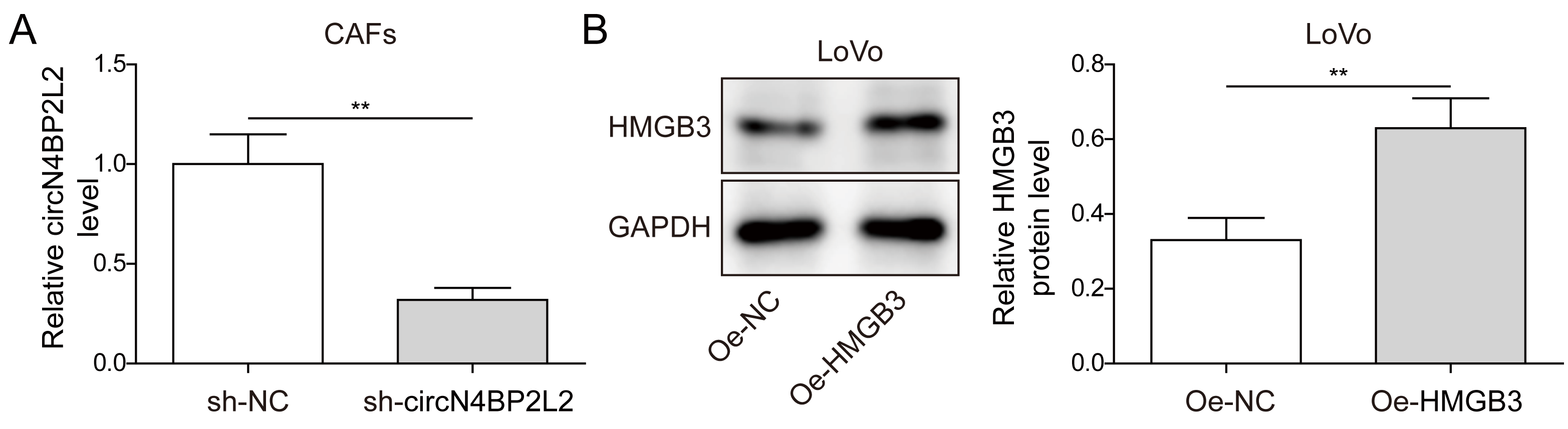

Supplement: Supplemental Material [file KCBT_A_2072164_SM5490.zip › figS1.tif]

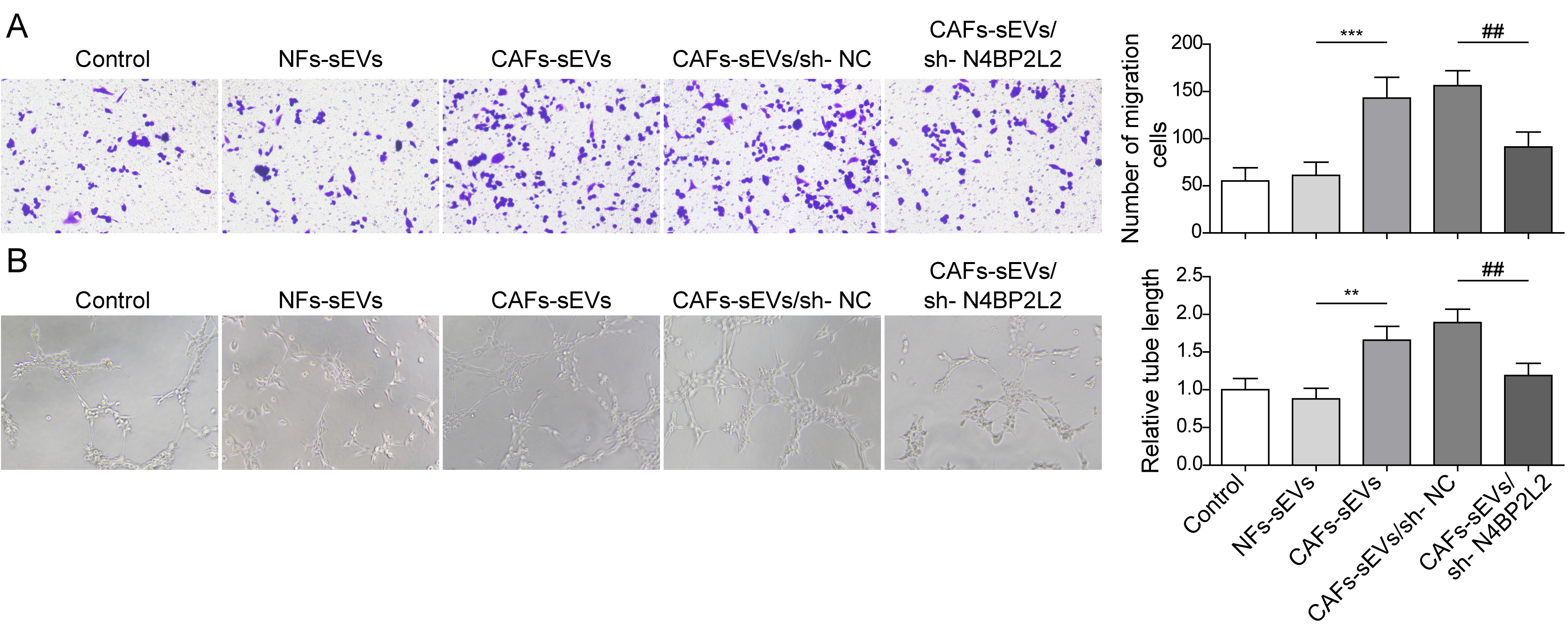

Supplement: Supplemental Material [file KCBT_A_2072164_SM5490.zip › figS2.tif]

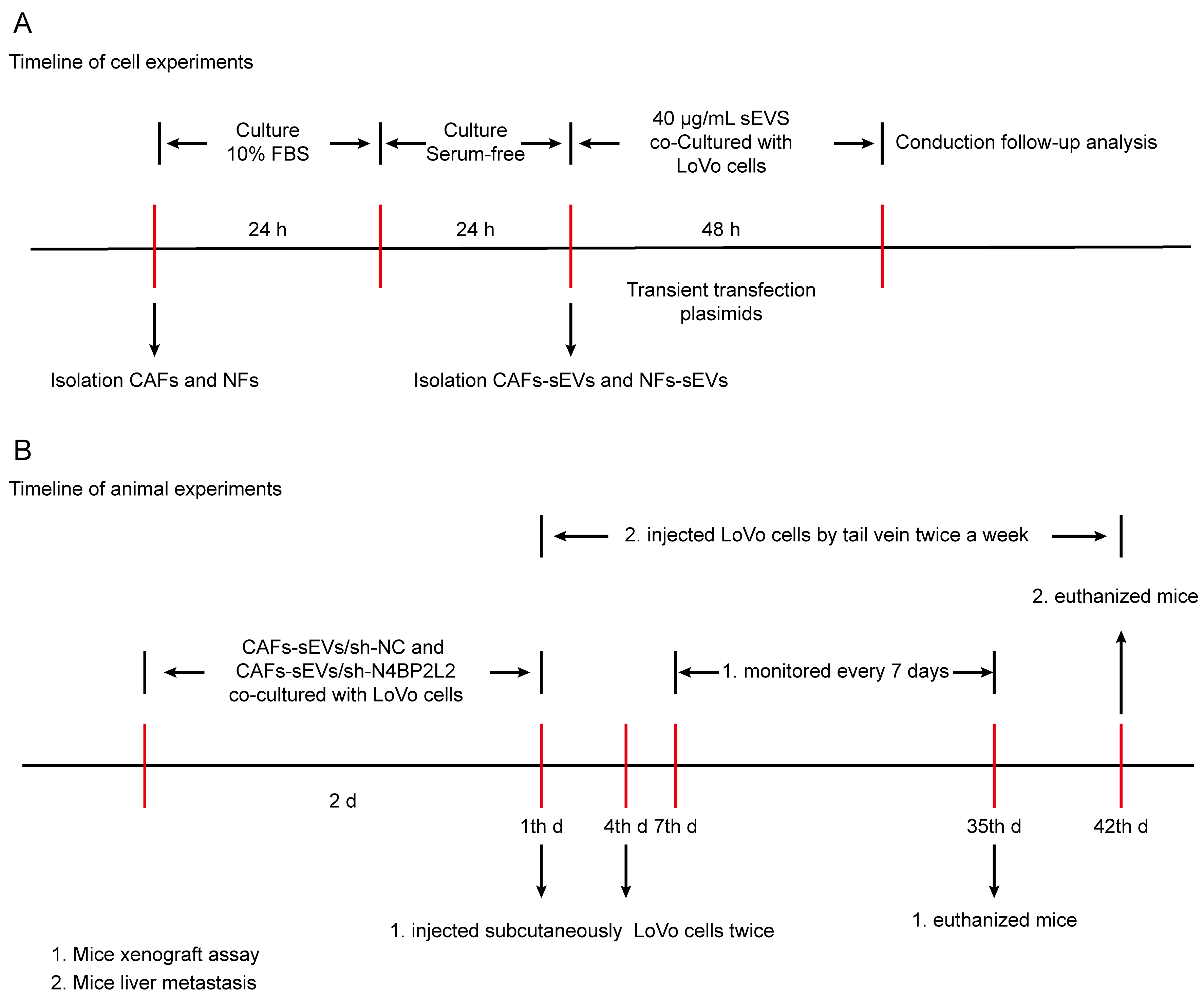

Supplement: Supplemental Material [file KCBT_A_2072164_SM5490.zip › figS4.tif]

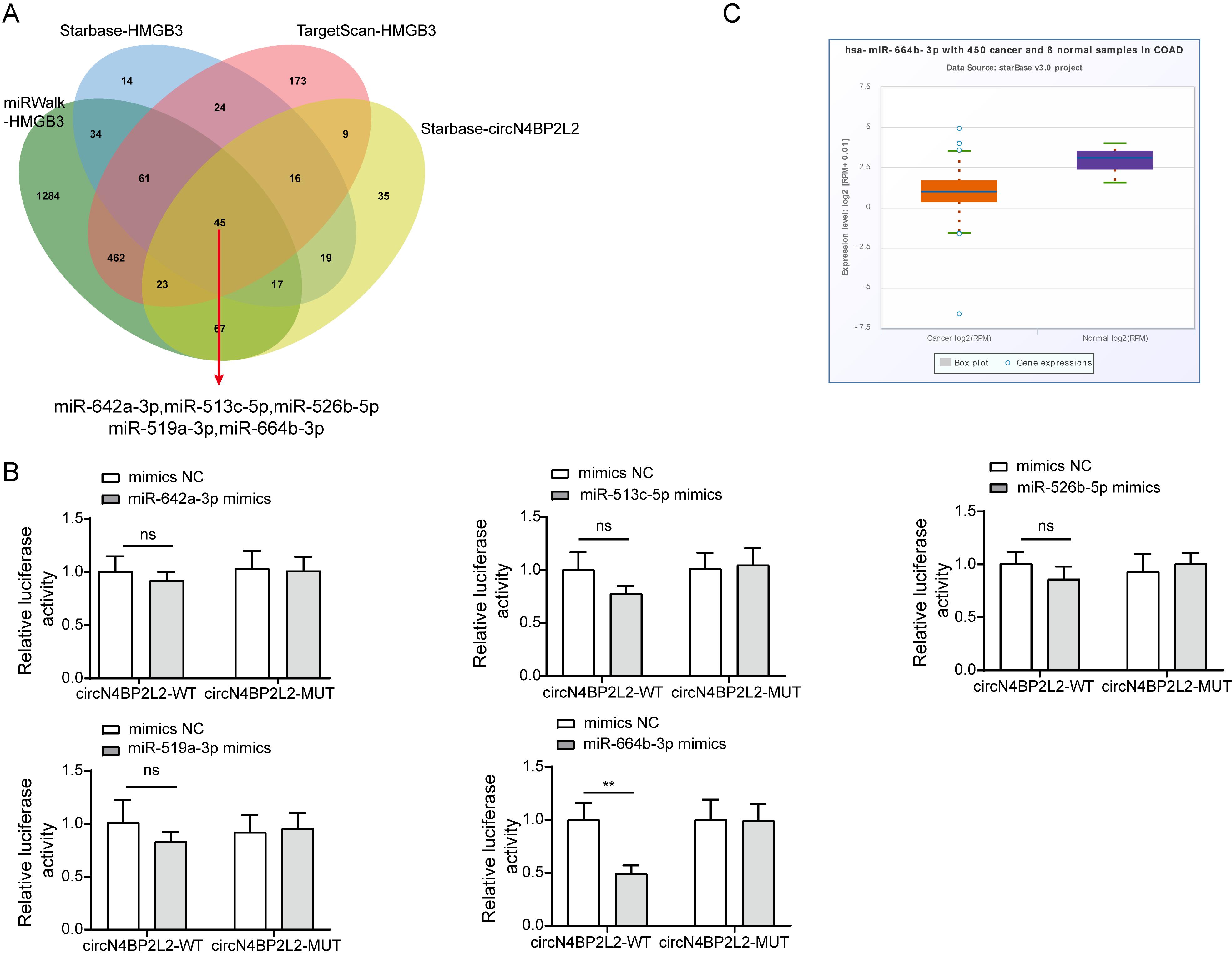

Supplement: Supplemental Material [file KCBT_A_2072164_SM5490.zip › revised figS3.tif]
